# Diffusion of individual birds in starling flocks

**DOI:** 10.1098/rspb.2012.2484

**Published:** 2013-04-07

**Authors:** A. Cavagna, S. M. Duarte Queirós, I. Giardina, F. Stefanini, M. Viale

**Affiliations:** 1 Istituto dei Sistemi Complessi, UOS Sapienza, CNR, via dei Taurini 19, 00185 Roma, Italy; 2 Dipartimento di Fisica, Università Sapienza, P. le Aldo Moro 2, 00185 Roma, Italy; 3 Institute of Neuroinformatics, University of Zurich and ETH Zurich, Winterthurerstrasse, 190 8057 Zurich, Switzerland

**Keywords:** collective behaviour, diffusion, self-organization

## Abstract

Flocking is a paradigmatic example of collective animal behaviour, where global order emerges out of self-organization. Each individual has a tendency to align its flight direction with those of neighbours, and such a simple form of interaction produces a state of collective motion of the group. When compared with other cases of collective ordering, a crucial feature of animal groups is that the interaction network is not fixed in time, as each individual moves and continuously changes its neighbours. The possibility to exchange neighbours strongly enhances the stability of global ordering and the way information is propagated through the group. Here, we assess the relevance of this mechanism in large flocks of starlings (*Sturnus vulgaris*). We find that birds move faster than Brownian walkers both with respect to the centre of mass of the flock, and with respect to each other. Moreover, this behaviour is strongly anisotropic with respect to the direction of motion of the flock. We also measure the amount of neighbours reshuffling and find that neighbours change in time exclusively as a consequence of the random fluctuations in the individual motion, so that no specific mechanism to keep one's neighbours seems to be enforced. On the contrary, our findings suggest that a more complex dynamical process occurs at the border of the flock.

## 1. Introduction

Self-organization and the spontaneous emergence of order in biological systems does not come much more spectacular than in large flocks of starlings (*Sturnus vulgaris*). At dusk, huge flocks move above the roost, exhibiting beautiful collective patterns. There is no leader in the group, and the collective movement is a unique consequence of local interactions between individuals [1,2].

A central question in collective animal behaviour is to understand what are the interaction rules through which global coordination emerges. For a long time, owing to the technical difficulties in reconstructing individual motion in large groups [3], data have been scarce. More recently, though, a new generation of experimental studies, both in two and in three dimensions, have been performed, establishing the basis for an empirically validated understanding of the interaction rules in collective animal behaviour [4–11]. What these data show is that several traits of collective motion are well reproduced by relatively simple models based on local interaction rules [12–18]. The fundamental ingredient shared by all models is the tendency of each individual to align to its neighbours. There is now a common consensus that this type of interaction is indeed a key aspect of collective motion in biology.

Alignment is a very important form of interaction in physics too: in ferromagnets, the tendency of each spin to align to its neighbours gives rise to a spontaneous global magnetization, much as a flock of birds develops a spontaneous global velocity. However, in adopting such a minimalistic approach to the description of flocks, not only does one make a gross oversimplification of the individual entities (birds are not spins, of course), but one also neglects a very fundamental difference between animal groups and spin systems: animals, unlike spins, each move with respect to another, so that the interaction network (i.e. who interacts with whom) changes in time. This crucial property of biological collective behaviour has a potentially large impact on how information propagates throughout the group.

Indeed, there are two mechanisms that contribute to the emergence of global coordination. The first one is the direct alignment of one individual with its interacting neighbours; from neighbour to neighbour, local ordering spreads over the interaction network to the whole group. This mechanism works even if individuals do not move with respect to each other, such as spins sitting on the sites of a crystalline lattice. The second mechanism, on the contrary, is intrinsically related to motion: when individuals move, two animals that were not directly interacting at a given time may become proximate neighbours and interact at a later time, so that information is more efficiently propagated throughout the group. It has been hypothesized that this mechanism reinforces correlations between individuals, strongly enhancing global ordering [19–21].

This extra ingredient of collective animal behaviour implies that we cannot simply investigate *static* aspects of the interaction network (such as, e.g. the number of interacting neighbours [7]), but we need to get information about the *dynamical* evolution of the interaction network. A first step in this direction is to study how individual animals move and rearrange within the group. This is what we do here for flocks of starlings in the field.

There are two other important reasons why it is relevant to have information about the relative dynamics of individuals. It has been found by Ballerini *et al*. [7], and later confirmed by Bialek *et al*. [18], that starlings in a flock interact with a fixed number of neighbours rather than with all neighbours within a fixed metric radius. This number is approximately seven [7,22]. A natural question is: what is the permanence in time of these seven individuals? Do they change uniquely due to the relative motion between individuals? Or is there any kind of relationship between interacting neighbours that keeps them together longer?

A second question concerns the border of the flock. Birds at the border are more exposed to predation than those at the interior. Former studies showed that the density of the flock at the border is larger than at the interior, probably as a consequence of the fact that border birds ‘push’ towards the inner part of the flock to get in [7]. Is there a border turnover? If so, how fast is it?

To quantify how individuals move through the group, we use a statistical perspective and adopt the powerful approach of diffusion processes [23,24]. To study diffusion, one needs not only the positions and velocities of the birds, but also the full individual trajectories. Individual tracking is a further level of difficulty with respect to static three-dimensional reconstruction (see §4), and a good performance is strictly related to having fast enough cameras and a large memory, in order to record long events. Even though this was not quite the case in our past experiments [6,7,25], we succeeded for a few flocking events, and for not too long a time interval, in retrieving a reasonable percentage of trajectories, with a sampling rate of 10 frames per second (table 1). Using these trajectories, we compute the diffusion properties of individuals with respect to the centre of mass and to neighbours. Moreover, we study the neighbour reshuffling rate and show how it is connected to the diffusion properties of individuals. Finally, we study the dynamics at the border of the flock.

**Table 1.** Details of the analysed flocks. The number of birds *N* is the number of individuals for which we obtained a three-dimensional reconstruction of positions in space (average over all frames). The duration *T* of the event is measured in seconds (calculated as number of frames × 10^−1^ s). *N*_LL_ indicates the number of retrieved trajectories that are as long as the entire time interval *T*. The last four columns give the values of diffusion and mutual diffusion parameters.

event	*N*	*T*(*s*)	*N*_LL_	*α*	*α*_*m*_	*D* (×10^−2^)	*D*_m_ (×10^−2^)
28-10	1246	1.5	785	1.83 ± 0.01	1.88 ± 0.02	3.8 ± 0.1	0.37 ± 0.04
48-17	871	1.6	350	1.73 ± 0.03	1.48 ± 0.02	3.5 ± 0.3	1.7 ± 0.03
49-05	797	1.6	146	1.71 ± 0.02	1.50 ± 0.02	3.9 ± 0.3	0.77 ± 0.06
58-06	442	3.1	140	1.69 ± 0.01	1.55 ± 0.02	3.6 ± 0.2	1.1 ± 0.04
69-09	239	4.6	62	1.64 ± 0.02	1.32 ± 0.01	4.1 ± 0.3	1.7 ± 0.04
69-10	1129	3.4	500	1.77 ± 0.02	1.72 ± 0.02	3.8 ± 0.2	0.89 ± 0.05

## 2. Results

### (a) Quantifying individual motion through diffusion

Why do individuals move through the flock and exchange positions? If each bird chose *exactly* the direction of motion and speed of its neighbours, then one would get a perfect flock where every individual keeps following the same direction as others. Relative positions would remain the same, defining an interaction network (who interacts with whom) that is fixed in time. However, imitation and mutual alignment are never complete, there is always an amount of uncertainty or arbitrariness in the individual choices. As a consequence, flight directions between neighbours are very similar, but not identical, differing by small ‘random’ fluctuations that flocking models usually describe through a stochastic noise term. Time after time, these fluctuations accumulate, determining a departure of the individual trajectories and a reshuffling of neighbourhood relationships. To describe such a process, it is useful to consider first the case where social forces are absent and individuals merely follow random moves. This is the renowned case of Brownian motion (where—originally—the random walkers were particles instead of birds). To quantify how much the Brownian walkers move in time, one can look at the average mean-square displacement as a function of time (i.e. at the average amount of distance travelled in a time *t*),2.1
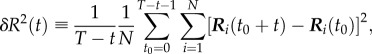
where 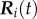 indicates the position of bird/particle *i* at time *t*, and where we have averaged over all *N* individuals in the group and over all time lags of duration *t* in the interval [0, *T*]. For Brownian motion, the mean-square displacement grows linearly with time [23] (i.e. 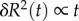), indicating that in their random wandering walkers depart increasingly from their origin. This behaviour, which is referred to as standard (or normal) diffusion, is rather robust and usually persists even in the presence of external forces or interactions between individuals. In some cases, however, such forces can enhance/deplete in a non-trivial way the effect of noise, leading to different diffusion laws. The majority of natural processes is well-described by a power-law dependence,2.2
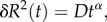
where *α*, the diffusion exponent, falls between 0 and 2, and *D* is the diffusion coefficient. The case *α* = 1 corresponds to Brownian motion and to normal diffusion. When *α* > 1, particles move/diffuse faster, and this is why this case is indicated as super-diffusive (the special case *α* = 2 corresponding to ballistic diffusion). Finally, we note that, although for very long times the type of diffusion is characterized by the value of the exponent *α*, for finite times even the value of the coefficient *D* plays a key role, larger values of *D* corresponding to more mobile particles/individuals.

### (b) Diffusion in the centre of mass reference frame

**Figure 1.**
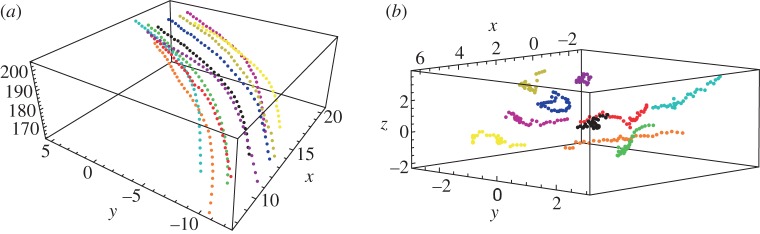
(*a*) Three-dimensional reconstruction of some trajectories of flock 69-10 (1124 individuals) in the laboratory reference frame. (*b*) The same trajectories in the centre of mass reference frame. All the axes are in metres.

Coming back to flocks, our aim is now to use the above definitions to quantify how much individuals move through the group and with respect to each other. Because flocks are strongly ordered, each bird moves predominantly in the same direction as the whole group. This contribution to individual motion is common to all birds and, if deviations were absent, would entail a fixed network of reciprocal positions. We are interested rather in what makes this network changing in time. Therefore, we need to take away this global component and focus on individual movements with respect to the flock's motion. This can be carried out by considering the birds' movements in the centre of mass reference frame: at each instant of time, the coordinates of an individual in this reference frame define its location inside the flock and, correspondingly, diffusion describes how much a bird has changed its position within the group (while at the same time co-moving with it). To visualize this point, in figure 1 we show a couple of trajectories of neighbouring birds, both in the camera's reference frame and in the flock's centre of mass reference frame. We notice that the centre of mass reference frame closely resembles the subjective perception individuals have of collective motion when flying together. Birds' individual velocities are in fact very close to that of the centre of mass (being the flock very polarized); therefore, the centre of mass frame is very similar to a frame co-moving with the birds themselves. An even more faithful representation of the individual perception (for a given bird) is provided by the mutual diffusion setting (see §2*c*).

To quantify diffusion behaviour in the centre of mass reference frame, we consider the mean-square displacement as in equation (2.1), but where coordinates are expressed in the centre of mass frame, such that2.3
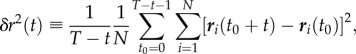
where 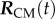 indicates the position of the centre of mass of the flock at time *t*, and 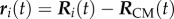 therefore represents the position of bird *i* in the centre of mass reference frame and 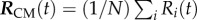 indicates the position of the centre of mass of the flock at time *t*. *N* is the number of birds in the flock and *T* the length of the time series.

**Figure 2.**
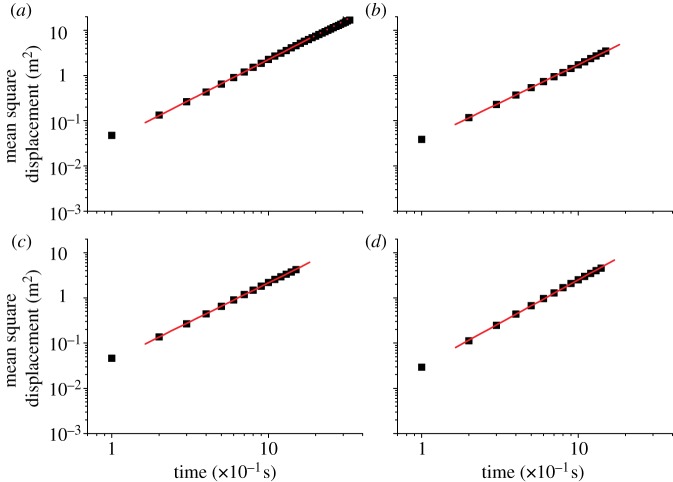
Mean-square displacement in the centre of mass reference frame, for four different flocking events: (*a*) 69-10, (*b*) 48-17, (*c*) 49-05 and (*d*) 28-10. Values of diffusion exponent and diffusion constant for each flock are: (*a*) *α* = 1.77 ± 0.02, *D* = 3.8 ± 0.02; (*b*) *α* = 1.73 ± 0.03, *D* = 3.5 ± 0.03; (*c*) *α* = 1.71 ± 0.02, *D* = 3.9 ± 0.03; and (*d*) *α* = 1.83 ± 0.01, *D* = 3.8 ± 0.01.

Using three-dimensional trajectories of individual birds in starling flocks, we computed the mean-square displacement following equation (2.3) for six flocking events (see §4). We find that diffusion of birds satisfies quite well the time dependence described by equation (2.2), with an exponent that is systematically larger than 1 (i.e*.* birds perform *super-diffusive* motion in the centre of mass reference frame). In figure 2, we present the data of four flocks, but results are similar in the other analysed flocks (table 1). Averaging the diffusion exponent over all the analysed events we get,2.4
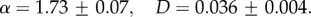

### (c) Mutual diffusion

The results discussed above indicate that individuals move within the group faster than Brownian walkers. This super-diffusive behaviour is probably the consequence of the interacting nature of collective motion, which gives rise to strong correlations between birds' flight directions. Velocity correlations were first studied for bird flocks by Cavagna *et al*. [25], where it was found that there are large correlated domains of birds with highly aligned velocities fluctuations. This means that if a bird is moving in a certain direction with respect to the centre of mass, then its neighbours will move along similar directions [25]. This fact suggests that diffusive displacement of a bird with respect to its neighbours should be smaller than with respect to the centre of mass. Is it so?

We can answer this question by calculating how much individuals in the flock move with respect to one another. We define an expression very similar to equation (2.3), but in which *mutual* mean-square displacement of birds *i* with respect to its nearest neighbour *j* at time *t*_0_ is considered,2.5
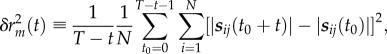
where 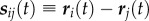 is the position of bird *j* (the nearest neighbour of *i* at time *t*_0_) in the reference frame of *i*. Also for mutual diffusion, we find a power-law behaviour,2.6
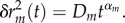


Averaging over all flocks, we obtain (table 1)2.7
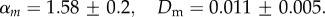


The representation of the time dependence of 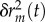 for the same flocks of figure 2 can be found in the electronic supplementary material, figure S1. From a comparison between the average parameters in equations (2.4) and (2.7), as well as the figures just mentioned, we can see that even though both diffusion and mutual diffusion have an exponent larger than 1, mutual diffusion is suppressed with respect to diffusion in the centre of mass. The available statistics do not allow us to conclude that the exponents are different (see the electronic supplementary material); however, in the intermediate time regime we are dealing with, this suppression is clear, especially looking at the diffusion coefficients. Mutual diffusion describes how, on average, an individual bird perceives the motion of its neighbours relative to its own. As we shall see, it is crucial to understand how neighbour reshuffling occurs in a flock.

### (d) Anisotropic diffusion

To push our analysis further, we can ask whether diffusion and relative motion occur isotropically or whether, on the contrary, privileged directions exist. The simplest way to probe the existence of privileged directions is to consider a matrix generalization of equation (2.3) (see the electronic supplementary material for details). If we diagonalize this matrix, the diagonal elements automatically provide the mean-square displacement along the principal axes of diffusion (see the electronic supplementary material, figure S3 for an example in four flocks). We can then compute, for each flock, the diffusion exponent and the diffusion coefficient along each axis. What we find is that diffusion is strongly anisotropic, occurring more strongly along certain directions (corresponding to larger diffusion exponents) than others. More precisely, the average diffusion exponents and coefficients along the three principal axes are
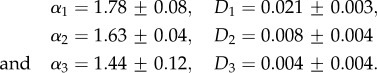


A previous study [6] showed that flocks tend to fly parallel to the ground, and therefore orthogonal to gravity. It is therefore natural to analyse the relation between the three principal axes of diffusion and the directions in space that are naturally relevant for a cruising flock, namely the direction of motion and gravity. To investigate this point, we computed the average (in time) scalar product of the three normalized eigenvectors of diffusion, **u**_1,_ **u**_2_ and **u**_3_, with the normalized vectors of the flock velocity (**u***_V_*) and of gravity (**u***_G_*). The results are the following:
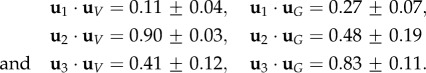


From these data, we see that gravity, **u***_G_*, has very high alignment with the direction of lowest diffusion, **u**_3_, whereas it has very low alignment with the direction of the largest diffusion, **u**_1_. The direction of global motion, **u***_V_*, has very high alignment with the second smaller diffusion direction, **u**_2_, whereas (like gravity) it has minimal alignment with the direction of largest diffusion, **u**_1_. We conclude that diffusion is suppressed along gravity and the direction of motion, whereas the axis of maximal diffusion, **u**_1_, is approximately perpendicular to both group velocity and gravity, and therefore it roughly coincides with the wings' axis.

The fact that diffusion along gravity is very limited is perhaps unsurprising, because of the energy expenditure that vertical motion requires. On the other hand, the higher weight of diffusion along the wings' direction versus the velocity direction is less obvious on a purely biological basis. As we shall see in §3, though, previous theoretical investigations indeed predicted that diffusion in flocking had to be much stronger along a direction orthogonal to the direction of motion, which is exactly what we observe here.

### (e) Neighbour reshuffling

A crucial consequence of motion and of mutual diffusion is that individuals may change their neighbours in time. Let us consider a (focal) bird *i* at an initial time *t*_0_ and its *M* nearest neighbours. After a time *t*, some of these *M* birds will not belong to the set of neighbours of *i* any more. To monitor how the neighbourhood changes, we can calculate the percentage of individuals that remain within the set of the *M* nearest neighbours of *i* after a time *t*. Let us therefore define the *neighbour overlap* as2.8
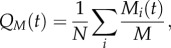
where *M_i_*(*t*) is the number of birds that are among the *M* nearest neighbours of bird *i* at both *t*_0_ and *t* + *t*_0_. The average runs over all the birds in the flock and over all initial times *t*_0_.

**Figure 3.**
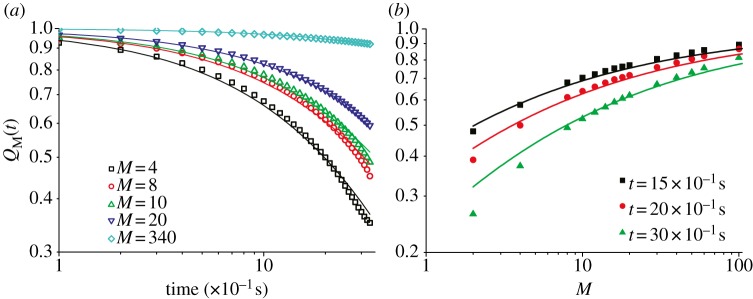
(*a*) Neighbour overlap *Q*_M_(*t*) versus *t*. (*b*) *Q*_M_(*t*) versus *M*. Full lines represent equation (2.9) with *c* = 0.048 (fitted value), whereas 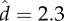 and *α* = 1.72 have the values predicted by the geometrical argument described in the electronic supplementary material. Data are for flock 69-10.

In figure 3, we show the evolution of the overlap, *Q_M_*(*t*), as a function of the time *t* and number of neighbours *M*. Clearly, if we set *M* = *N* (i.e*.* if we choose a neighbourhood as large as the whole flock) then the overlap remains by definition constant and equal to 1. When *M* < *N*, we see that the overlap smoothly decreases in time owing to birds' motion. We conclude that neighbour reshuffling *does* happen, even for very close neighbours. This implies that the interaction network is changing in time and that there is no indication of a preferred structure of neighbours in the flock. We also notice, however, that the process of reshuffling of the neighbours occurs on a time-scale of a few seconds, which is rather long. We will analyse the implications of this fact in §3.

Interestingly, it is possible to explain the behaviour of the cluster overlap purely in terms of the diffusion properties described in the previous sections. The basic idea is simple: consider a focal bird, and its neighbourhood of *M* birds. We ask how many neighbours the focal bird can lose in a time *t*. The most at risk are those in the outer edge of the neighbourhood. We make the very crude approximation that in a time *t* the outer birds will have travelled a distance 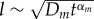, which is a sort of deterministic interpretation of mutual diffusion equation (2.6). In this case, the number of lost neighbours will be of the order 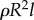, where *R* is the radius of the neighbourhood, which is connected to *M* by the simple relation 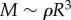. Using this argument, we finally get2.9
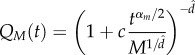
(see the electronic supplementary material for the details), where *c* is a constant related to the flock density *ρ* and to the mutual diffusion coefficient *D_ij_* (see the electronic supplementary material). For infinite and homogeneous flocks, 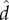 coincides with the space-dimension, 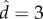, whereas for finite flocks, owing to the presence of the border, we have an effective dimension 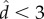. The value of 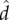 can be fixed by making a power law fitted to the formula 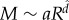 (see the electronic supplementary material, figure S2). We find 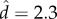.

Using the value of *α_m_* obtained in §2*c*, we get a very good agreement with the data (figure 3), both for what concerns the dependence of *Q* on *t* and on *M*. Such agreement indicates that neighbour reshuffling is entirely ruled by diffusion: there seems to be no ad hoc mechanism used by birds to pick up their neighbours, nor any specific attempt to keep them fixed in time. Rather, neighbour reshuffling is simply the result of diffusion taking its course, so that at each instant of time, each bird is interacting with whichever birds have been brought there by their super-diffusive wandering throughout the flock.

### (f) Permanence on the border

**Figure 4.**
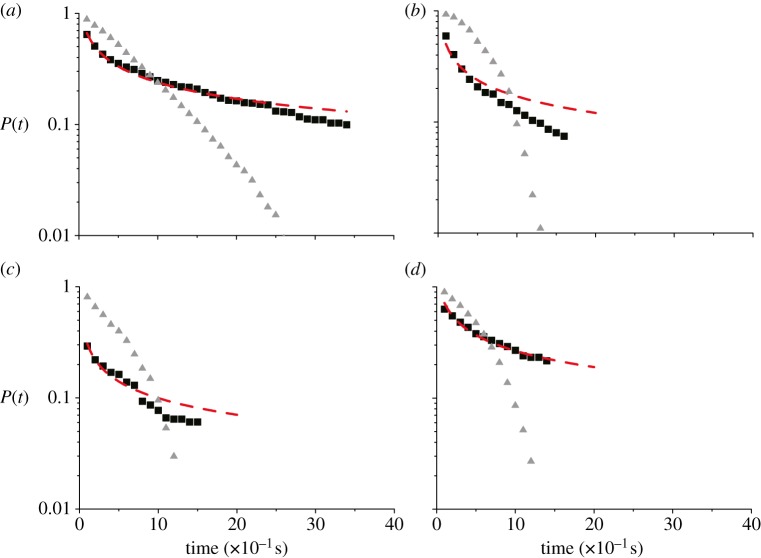
Border survival probability *P*(*t*) (black squares) for four different flocking events: (*a*) 69-10, (*b*) 48-17, (*c*) 49-05 and (*d*) 28-10. The dashed red line corresponds to a fit of the data using a Brownian functional form. For each flock we also report the survival probability for internal individuals (grey triangles).

Because of the attacks of predators and of possible interactions with other external perturbations, birds at the border of the flock might exhibit specific dynamical properties [26]. To investigate this issue, we calculate the border survival probability, *P*(*t*), defined as the probability that a bird initially at the border remains on the border for a time greater than *t* (for a precise definition of the flock's border, see electronic supplementary material). The data for *P*(*t*) are shown in figure 4 for four different flocks.

Given our success in explaining neighbour reshuffling purely by using the diffusion properties, it is interesting to ask whether the border survival probability too is ruled simply by diffusion or whether there is some extra dynamical ingredient ruling the way birds remain on the border. We may start by saying that once a bird has travelled more than the average distance *l*_B_ between border and first internal nearest neighbour, it has left the border. If we use the same crude approximation as for neighbour reshuffling, namely that in a time *t* a bird travels on average a distance 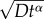, we can get an estimate of the time-scale birds remain on the border,2.10
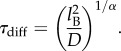


Using the measured values for flock 69-10 for *l*_B_, *α* and *D*, this gives *τ*_diff_ = 0.8 s. On the other hand, if we look at the data in figure 4 we can see that after a fast and short initial decay, then the curve exhibits a rather long tail, indicating that persistence on the border can in fact be much longer. Indeed, a simple exponential fit of flock 69-10 gives a time-scale *τ* = 2.5 s, three times larger than what mere diffusion predicts. A similar underestimation occurs for the other flocks. It seems that a naive diffusion argument does not work and that individuals at the border tend to exchange positions with neighbours less than internal individuals do.

The discrepancy between internal and border dynamics is confirmed by comparing the border survival probability with the analogous survival probability for internal birds. In figure 4, together with the border *P*(*t*), we also plot the probability that in a time *t* an internal bird remains within a distance *l*_B_ from its initial position in the centre of mass reference frame (i.e. the probability that a positional swap with a neighbour does not occur). This internal survival probability decays much faster than the border one. Analytic computations allow calculation of rigorous bounds for the survival probability of a super-diffusive walker with diffusion exponent *α* (see electronic supplementary material) [27]. The empirical *P*(*t*) for internal birds is fully consistent with these predictions, confirming once again that mutual rearrangements of internal individuals can be explained in terms of a diffusion mechanism. However, the same is not true for the border survival probability.

Why, then, do birds on the border tend to exchange position with neighbours less than internal birds do? First, and most trivially, we need to consider that border individuals—due to their peripheral location—lack neighbours on one side. Therefore, any movement larger than *l*_B_ but in the outward direction does not decrease the relative distance with any internal neighbour and leaves the bird on the boundary of the flock. To take this into account, the appropriate quantity to look at is therefore the probability that an individual moves less than *l*_B_ only in the inward direction. For a generic diffusion process, there are not analytic expressions for this quantity. However, in the case of a Brownian walker, the computation can be easily carried out [23], leading to2.11
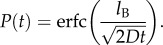


A fit of the data with this Brownian functional form is displayed in figure 4. While this fit captures the convex shape of the curve, the empirical border survival probability systematically decays faster for times larger than the typical diffusive scale (2.10). This can be explained by noticing that even if border birds can arbitrarily move towards the outside while still remaining peripheral, they in fact never increase their distance from the flock by too much, as this would imply leaving the group and losing altogether the benefits of the collective motion. It is better to be in the border than to go astray [28]. In getting equation (2.11), however, all these large outer ‘walks’ are considered as possible positive contributions to the permanence on the border at large times. Thus, we must expect the real survival probability (which does not include such walks) to be smaller than the one predicted by equation (2.11), as we indeed find.

Finally, there might be an additional effect to be taken into account. Previous experimental observations show that the density of flocks is larger at the border than at the interior [7]. This effect could be a consequence of the fact that birds compete with each other for a place in the interior of the flock. This struggle for the occupation of the same internal space would imply that when attempting to move inward, a border bird experiences an outward repulsion produced by its internal neighbours, pushing it outside again. The attempt to move inward is then reiterated, until by some fluctuation the bird successfully leaves the border. This mechanism clearly contributes to increase the survival probability of border birds when compared with internal ones.

## 3. Discussion

Our results show that diffusion in the centre of mass reference frame occurs with an exponent of *α* = 1.73, much larger than the Browninan case (*α* = *1*). Birds within a flock are therefore strongly super-diffusive. How do theoretical predictions of flocking diffusion compare with our data? Hydrodynamic theories of flocking [19,20,29] make some predictions about the emergence of anomalous diffusion. In particular, in two dimensions, these theories predict super-diffusive behaviour, with an exponent *α* = 4/3 [29]. Numerical simulations in two-dimensional models of self-propelled particles support these predictions [29,30]. However, these predictions have been made for two-dimensional systems, whereas our data are three-dimensional. Hydrodynamic predictions in three dimensions are much harder to perform, but according to a conjecture put forward by Toner & Tu [20], it would be expected that *α* = 1 in three dimensions, in contrast to our result. On the other hand, numerical simulations in three dimensions [31] give *α* = 1.7, in agreement with our experimental value. We believe that, now that experimental data about diffusion are available, both theoretical and numerical studies in three dimensions should be reconsidered more carefully, as the prediction of the right diffusion properties can be a very effective model selection tool.

Our diffusion data display strongly anisotropic behaviour. Motion is quite limited in the plane formed by flock velocity and gravity, while it is much stronger along a direction perpendicular to that plane. We can roughly identify this direction of maximal diffusion with the wings' axis. There is a compelling geometric argument to explain the origin of anisotropic diffusion [20]: if birds make small errors *δθ* in their direction of motion, then their random displacement perpendicular to the mean direction of motion 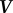 is much larger than that along 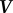; the former is proportional to 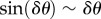, whereas the latter is proportional to 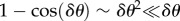. Therefore, diffusion is suppressed along the direction of motion 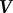. This simple argument does not take into account the role of gravity, which has the effect of further depressing vertical diffusion on the plane perpendicular to 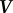. As a consequence, one expects to have minimal diffusion along both 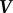 and gravity, and maximal diffusion along the direction perpendicular to them. This is exactly what we find.

When we consider mutual diffusion, namely how much a bird moves with respect to its nearest neighbours, we find diffusion exponents similar to diffusion in the centre of mass reference frame, but much lower diffusion constants. In other words, birds move *less* with respect to their neighbours than with respect to the centre of mass. This fact is the consequence of the very strong and long-ranged spatial correlations of the velocity fluctuations observed by Cavagna *et al*. [25]. Neighbouring birds' displacements in the centre of mass reference frame are similar, so that birds do not depart from each other as much as they move throughout the flock.

From the full individual trajectories, we calculated the neighbour overlap *Q_M_*(*t*) and thus quantified how much, on average, the local neighbourhood of a focal bird changes in time. Our data show that neighbour reshuffling occurs, so that each bird gradually changes all its interacting neighbours over time. There is no indication of a fixed structure of neighbours in the flock. In fact, we showed that a very simple model, whose only ingredient is mutual diffusion, reproduces quantitatively well the neighbour overlap, without the need of any extra dynamical ingredients. This fact seems to indicate that the neighbours each bird is interacting with at each instant of time are not selected on the basis of a biological criterion; they just randomly happen to be there, according to diffusion laws.

Even though neighbour reshuffling definitely occurs, it seems *not* to be a very fast process. To give full validity to such a statement we should define a time-scale (the birds' ‘clock’), which is not straightforward. Still, we do expect any kind of update of the internal state of motion of a bird to happen on a rather fast time-scale (let us say definitely smaller than 0.1 s). Hence, the fact that, for example, it takes about 3.5 s to change only half of 10 neighbours (figure 3) really seems to indicate that neighbour permanence is rather high. This is interesting. Indeed, according to several theoretical and numerical studies, the fact that the interaction network changes in time has the effect of reinforcing the alignment order in the flock [19,32,33]. Changing the neighbours over time amounts to having an *effective* number of interacting neighbours that is larger than the instantaneous one.

However, there may be a trade-off: exchanging neighbours *too* quickly could be detrimental for establishing long-range order in the flock. At each time-step, one individual tries to align its velocity to that of its neighbours; but there is noise, so that alignment is not perfect and it may take several time-steps to consolidate consensus. If, however, the pool of neighbours changes completely from one time-step to the next, it will be very hard to beat noise and therefore to dynamically reach global consensus. If a trade-off exists, there should be an optimal neighbour reshuffling rate that makes global order easiest to achieve at the dynamical level. However, even if an optimum exists, then it does not imply that the natural system is actually at the optimum. The comparison of theoretical models, where the rate of neighbour reshuffling can be artificially altered, with our experimental data (which give quantitative substance to these speculations), can help understanding of whether an optimum neighbour reshuffling exists and to what extent natural flocks of birds are close to such an optimum.

Finally, we have investigated the dynamics of individuals at the border of the flock. What we find is an intriguing difference between motion within the flock and motion at the border. The survival probability of individuals at the border is indeed significantly larger than the survival probability of internal individuals: birds stay on the border longer than the way internal birds keep their position inside the flock. Our analysis suggests that in doing this, individuals on the border balance the tendency to exchange neighbours owing to motion, the availability of void space outside the flock and the reluctance of internal neighbours to give up a more favourable position.

When a predator (such as the peregrine falcon) attacks a flock, it is mostly birds on the border that get captured. Hence, the border is a dangerous place. And yet, bird dynamics do not accelerate border turnover. It seems that the flock self-organizes out of the individual selfish tendency not to stay at the border. This situation is reminiscent of the ‘selfish herd’ scenario described by Hamilton [26]. Border dynamics is very fascinating and very important, and we have just started scratching the surface of it. New data (and more specifically longer and more exhaustive trajectories) are needed to be able to fully unveil border dynamics.

## 4. Methods

Analysed data were obtained from experiments on large flocks of starlings (*S. vulgaris*), in the field. Using stereometric photography and computer vision techniques [8,33], the individual three-dimensional coordinates were measured in groups of up to a few thousands individuals [6–8]. For a number of flocking events (table 1), we could retrieve individual trajectories. Each event consists of up to 40 consecutive three-dimensional configurations (individual positions), at time intervals of 0.1 s. We developed a tracking algorithm that connects the three-dimensional spatial positions of the *same* individual through time. Temporal matching between consecutive times is based on a patter algorithm of the same kind as the one used to solve the stereometric matching (see [8]). This two-time match is effective but never complete. At each instant of time, a small percentage of individuals (typically below 5% in our case) is not reconstructed owing to occlusions on the images and segmentation errors. Owing to this, a mere iteration of two-time matches only brings a set of very short interrupted trajectories. To overcome this problem, we developed a Monte Carlo algorithm that allows for ‘ghosts’ to simulate the occurrence of missing three-dimensional reconstructions, and patches together pieces of trajectories by optimizing an appropriate measure combining average smoothness, three-dimensional constraints and number of ghosts. Thanks to this algorithm, we could retrieve a reasonable percentage of individual trajectories as long as the entire event.

Given a flocking event, we considered the subset of retrieved long-lasting trajectories and computed the mean-squared displacement and mutual square displacement, following equations (2.3) and (2.5). To estimate the diffusion exponents and coefficients, we fitted the resulting time dependence in log–log scale between time lags of 0.4 and 1.5 s, which takes into account the length of all the data at our disposal. Results for the individual flocks are reported in table 1, and correspond to super-diffusive behaviour. The statistical significance of this finding in connection with the finiteness of the time series was tested using synthetic data (see the electronic supplementary material for a full account of the procedure).
